# Preparation of Nanofibers Mats Derived from Task-Specific Polymeric Ionic Liquid for Sensing and Catalytic Applications

**DOI:** 10.3390/polym13183110

**Published:** 2021-09-15

**Authors:** David Valverde, Iván Muñoz, Eduardo García-Verdugo, Belen Altava, Santiago V. Luis

**Affiliations:** 1 Departamento de Química Inorgánica y Orgánica, Universidad Jaume I, Avda. Sos Baynat, s/n, 12071 Castelló de la Plana, Spain; dvalverdeb@uned.ac.cr (D.V.); munozi@uji.es (I.M.); altava@uji.es (B.A.); luiss@uji.es (S.V.L.); 2 Vicerrectoría de Investigación, Universidad Estatal a Distancia, Heredia 40205, Costa Rica

**Keywords:** poly(homocysteine thiolactone), nanofibers, electrospinning, sensing, catalysis

## Abstract

Nanofibers mats derived from the task-specific functionalized polymeric ionic liquids based on homocysteine thiolactone are obtained by electrospinning them as blends with polyvinylpyrrolidone. The presence of this functional moiety allowed the post-functionalization of these mats through the aminolysis of the thiolactone ring in the presence of an amine by a thiol–alkene “click” reaction. Under controlled experimental conditions the modification can be performed introducing different functionalization and crosslinking of the electrospun fibers, while maintaining the nanostructure obtained by the electrospinning. Initial studies suggest that the nanofibers based on these functionalized polymeric ionic liquids can be used in both sensing and catalytic applications.

## 1. Introduction

Electrospinning techniques combine simplicity, versatility, and low cost with superior capabilities to elaborate scalable ordered and complex nanofiber (NFs) assemblies [1]. NFs mats present advantages over other nanostructured materials due to their high porosity, high surface-area-to-volume ratio, interconnectivity, and ease preparation [2]. These have demonstrated their potential application in different fields such as filter media [3], oil/water separation [4], energy [5], drug delivery [6], sensors [7,8], food packaging [9], tissue engineering [10], catalysis [11], etc.

Polymeric Ionic Liquids (PILs) merge the unique properties of the Ionic Liquids (ILs) with those of advanced materials [12,13]. These macromolecules formed by ionic liquid units connected through a polymeric backbone present an enhanced mechanical stability, processability, flexibility and durability, and improved dimensional control over their structure [14]. PILs with different architectures and properties can be designed according to the desired specific application [15,16]. The combination of the unique properties of the PILs and the use of the electrospinning can lead to nanofibers mats for different applications. However, there are only few examples of the preparation of NFs derived from PILs [17]. In this regard, the molecular structural diversity introduce for PIL has been exploited to modify surface wetting properties of the mats for oil/water separations [18].

PILs nanocomposite fibrous membrane containing metal nanoparticles of tungsten oxide fabricated by electrospinning has been used for the preparation of functional electrolyte for quasi solid-state dye sensitized solar cell (QSS-DSSC) [19]. The power conversion efficiency and stability were improved using electrospun PILs nanofiber composite when compared to the cell with the liquid electrolyte. In a similar way, PILs obtained by chemical modification of poly(vinylidene fluoride-co-hexafluoropropylene) with different molar ratios of 1-butylimidazolium iodide has been explored for the preparation QSS-DSSC [20]. The membrane obtained by electrospinning showed good efficiency with a high *η* of 9.26%, and excellent long-term stability (up to 97% of its initial *η* over 1500 h). PILs with well-defined chemical structure were also applied as precursors for nitrogen-doped carbon NFs mats via the electrospinning technique [21], The electrical conductivity of the mat was ca. 200 S cm^−1^, which is fairly satisfactory compared to carbons prepared from many other organic polymers and materials at the same temperature.

**Figure 1:**
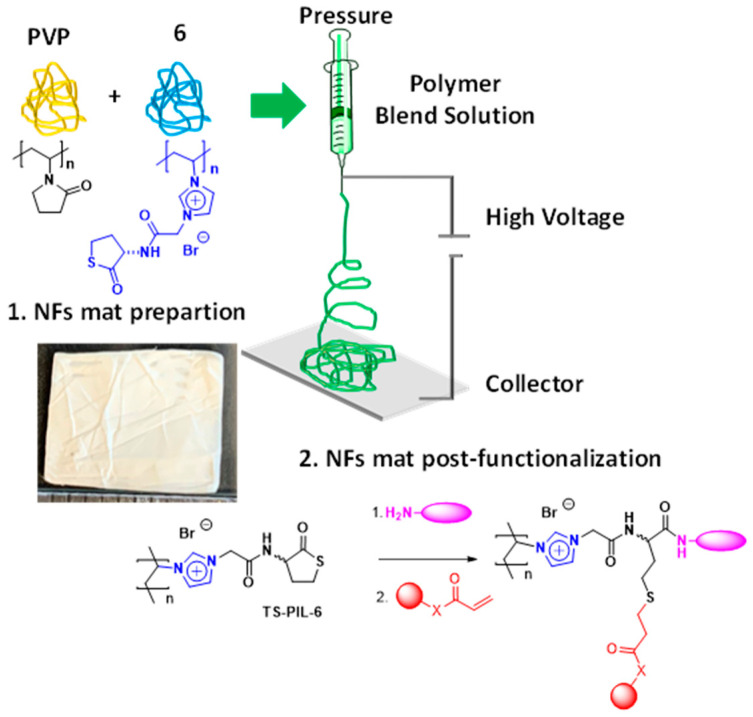
Preparation and post-functionalization of NFs mats derived from TS-PIL **6**.

Here, we report on the use of functionalized PILs with amide-thiolactone moieties as a unique platform for the development of NFs mats by electrospinning (Figure 1). The presence of the thiolactone unit allows the orthogonal post-functionalization of these NFs mats through its aminolysis in the presence of an amine (first level of functionalization) followed by the so-called thiol–alkene “click” reaction of the generated –SH groups in the presence of an alkene (second level of functionalization).

The high flexibility of this post-modification protocol allows one to obtain a large variety of TS-PILs through the synthetic manipulation of a reduced number of common simple intermediates, easily available even in large scale. The methodology here reported not only allows introducing orthogonal functionalities but also provides a simple strategy for crosslinking the NFs without losing their NFs morphology. The simple preparation and post-medication procedures here developed allows exploiting these NFs mats as advanced materials for sensing and catalytic applications.

## 2. Materials and Methods

### 2.1. Materials

All the reagents and solvents used were commercially available. K_2_CO_3_ (Scharlau extra pure, Barcelona, Spain), *dl*-homocysteine thiolactone hydrochloride (Aldrich 99%), bromoacetyl bromide (Aldrich 98%, St. Louis, MO, USA), citric acid (Aldrich 99%), anhydrous MgSO_4_ (Scharlau extra pure), 4,4’-azobis(2-methylpropionitrile) (Aldrich 98%), poly(4-vinylpyridine) (Aldrich, average Mw~160,000 g/mol), and were used as received. The 1-vinylimidazole (Aldrich 99%) was purified by vacuum distillation. All solvents used were of analytic degree.

### 2.2. General Characterization Protocols

ATR-FTIR spectra were obtained using a spectrometer (JASCO FT/IR-6200) equipped with an ATR (MIRacle single-reflection ATR diamond/ZnSe) accessory at 4 cm^−1^ resolution (4000–600 cm^−1^ spectral range). ^1^H-NMR experiments were carried out using a Varian INOVA 500 (^1^H-NMR, 500 MHz, ^13^C, 125 MHz) and Bruker, Billerica, MA, USA (^1^H-NMR, 400 MHz) spectrometer.

### 2.3. Synthetic Protocols

#### 2.3.1. Synthesis of Thiolactone Derivative (**3**)

*dl*-homocysteine thiolactone hydrochloride (9.2 g, 60 mmol) was dissolved in milli-Q H_2_O (20 mL) in a round-bottom flask equipped with a stir bar an ice-cooled (0 °C), then K_2_CO_3_ (25,7 g, 186 mmol) was slowly added. The resulting mixture was stirred for 5 min and CH_2_Cl_2_ (200 mL) was added. After this, bromoacetyl bromide (10.5 mL, 120 mmol), dissolved in CH_2_Cl_2_ (40 mL), was added dropwise during 1 h. After the addition, the reaction mixture was allowed to react for 30 min at 0 °C and then 1 h when the reaction reached rt. A 5% solution of citric acid (2 × 40 mL) was added afterwards to the reaction mixture. The organic phase was decanted, washed with milli-Q^®^ H_2_O (2 × 40 mL), dried over anhydrous MgSO_4_, filtered, and concentrated providing a white solid used without further purification. Yield: 70%. ^1^H NMR (CD_3_CN, 400 MHz): δ (ppm): 7.03 (s, 1H), 4.65–4.48 (m, 1H), 3.85 (s, 2H), 3.45–3.21 (m, 2H), 2.58 (dddd, 1H), 2.24–2.06 (m, 1H). ^13^C NMR (CD_3_CN, 101 MHz): δ (ppm): 205.7, 167.4, 59.9, 31.1, 29.4, 27.9.

#### 2.3.2. Synthesis of Poly(1-vinylimidazole) (**5**)

**Scheme 1:**
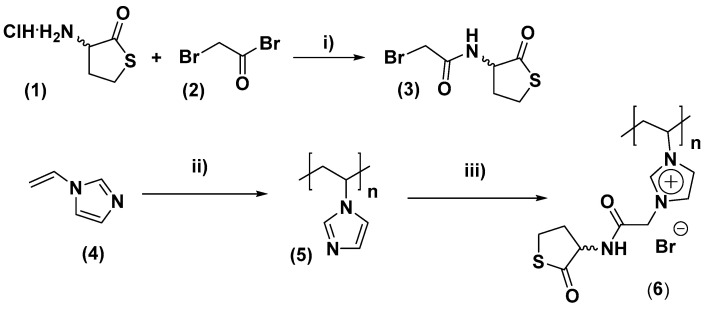
Synthesis of PIL **6**. (**i**) K_2_CO_3_, H_2_O/CH_2_Cl_2_ 0 °C, then rt. (**ii**) AIBN, 100 °C, 24 h DMF. (**iii**) DMF, 80 °C, 24 h.

Compound **5** was obtained as shown in Scheme 1 according to methods already reported in the literature. Characterization data were consistent with published values [21].

#### 2.3.3. Quaternization of Poly(1-vinylimidazole) (**5**) to Produce (**6**)

Poly(-1-vivylimidazole) (**5**) (5.9 g, 5.88 mmol) was dissolved in dry DMF (15 mL). The solution was heated to 85 °C, and **3** (2,1 g, 8.82 mmol) dissolved in dry DMF (15 mL) was added dropwise. The reaction mixture was bubbled by N_2_ and was allowed to react for 24 h, to 85 °C with stirring keeping N_2_ atmosphere. After cooling down to room temperature the polymer was precipitated in to 1 L of diethyl ether and the mixture was left stirring for 30 min. Finally, the diethyl ether was decanted and the solid was washed with diethyl ether (4 × 20 mL) and dried under high vacuum. The solid was purified by dialysis in water and lyophilized, obtaining a brown solid. Yield: 40%. ^1^H NMR (400 MHz, D_2_O) δ (ppm): 7.58 (d, 1H), 5.11 (s, 1H), 4.36 (s, 1H), 3.51 (d, 1H), 2.55 (d, 2H). ^13^C NMR (101 MHz, D_2_O) δ (ppm): 209.93, 166.27, 137.02, 136.21, 126.40, 119.17, 59.70, 51.16, 39.50, 29.70, 27.84.

### 2.4. Preparation of the Electrospun Fibers

The electrospinner used was a Fluinateck LE 100.V1 of BioInicia, Paterna, Spain. The polymer solution was loaded into a plastic syringe equipped with a stainless steel needle tip of 0.9 mm inner diameter. The tip-collector working was fixed at x: 15.5 cm, y: 15.5 cm, z: 20.0 cm. The applied voltage was kept between +19.5 and −14.7 kV and the flow rate was 1500 μL min^−1^. The electrospun fibers were collected on aluminum foil that covered a rotating collector (100 rev min^−1^). All experiments were carried out at room temperature and relative humidity between 39 and 41%. The obtained membranes were vacuum dried overnight in an oven at 50 °C.

## 3. Results and Discussion

### 3.1. Synthesis and Characterization of a Task Specific PIL Containing Thiolactone Fragments

The desired functionalized PIL containing amino-homocysteine thiolactone units was obtained as depicted in Scheme 1. TS-PILs **6** was synthesized by alkylation of poly(1-vinylimidazole) (**5**) with the 2-bromoacetamide of the amino-homocysteine thiolactone **3** in DMF. Poly(1-vinylimidazole) (**5**) was prepared following the methodology reported by Yuan and co-workers [21]. The alkylating agent could be synthetized in multi-gram scale by reaction of dl-homocysteine thiolactone hydrochloride (**1**) with 2-bromoacetyl bromide (**2**). This methodology allows for the synthesis of functionalized PILs with high molecular weight, which are difficult to access by direct polymerization of the corresponding functionalized IL-monomers. Indeed, it should be mentioned that all attempts to obtain the polymer **6** by direct polymerization of the IL-monomer resulting from the alkylation of vinyl imidazole with **3** were unsuccessful.

**Figure 2:**
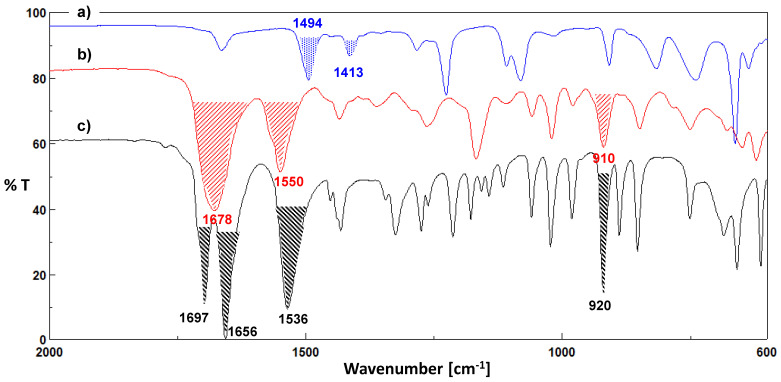
Synthesis of the polymer **6** monitored by ATR-FTIR. (**a**) Polymer **5**, (**b**) Polymer TS-PIL **6** and (**c**) alkylating agent containing amino-thiolactone units (**3**).

The chemical modification of the polymer **5** was confirmed by ATR-FTIR and NMR analysis. Figure 2a shows the C=C/C=N stretching bands at 1484 cm^−1^ and 1440 cm^−1^ assignable to the presence of the imidazole rings in polymer **5**. The disappearance of these bands suggests the complete conversion of these imidazole rings into the corresponding imidazolium salts (Figure 2b).

The IR-spectra of the polymer **6** also showed the appearance of a broad band at 1650–1720 cm^−1^ associated to the C=O stretching of both thiolactone and amide carbonyl groups, appearing as two different bands for **3** (1697 cm^−1^ and 1656 cm^−1^, respectively). The polymer **6** also showed an intense peak at 910 cm^−1^ assignable to the C-S stretching of the thiolactone ring.

**Figure 3:**
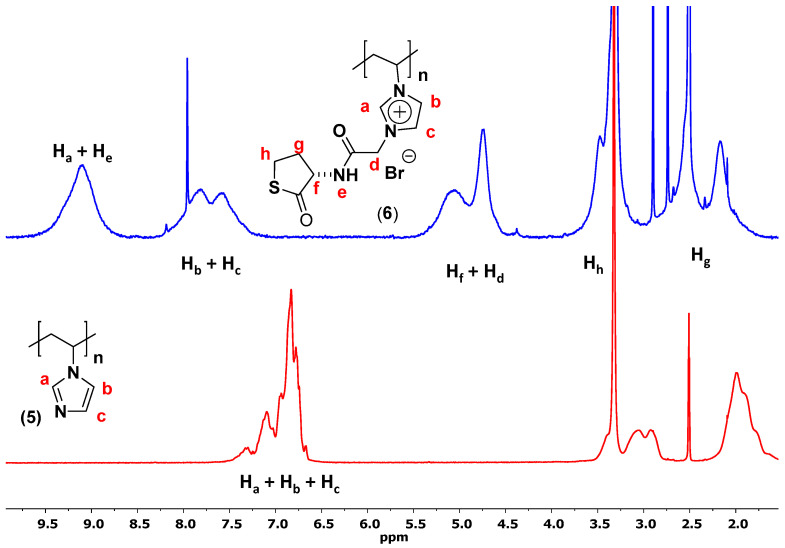
^1^H-NMR (D_2_O) spectra of **5** (poly(1-vinylimidazolium bromide)) and **6** (3-acetamide- *dl*-homocysteine thiolactone-1-vinylimidazolium bromide).

The ^1^H-NMR of polymers **5** and **6** confirmed a quantitative quaternization of the imidazole rings (Figure 3). The proton signals of the imidazole ring in **5** (6.4–7.5 ppm) disappeared after the alkylation and shifted to 7.5–9.5 ppm in good agreement with the position expected for imidazolium signals [21]. A series of new signals corresponding to the protons of the -CH_2_- and –CH groups in the thiolactone units appeared at 4.7–5.4 ppm. The integrals for the thiolactone proton signals at 4.7–5.4 ppm and the imidazolium and amide proton signals (7.5–9.5 ppm) displayed the expected ratio of 4.0/3.0. The presence of the peak at 209 ppm associated with C=O of thiolactone and the peak at 166 ppm assignable to the C=O of the amide on the ^13^C-NMR of polymer **6** confirmed successful alkylation of the polymeric imidazole (Figure S14).

### 3.2. Synthesis of NFs Mats by Electrospinning

The preparation of NFs mats was evaluated using different electrospinning parameters, which could be adjusted during the process by collecting small samples in-situ and analyzing their morphology by optical microscope. The Table S1 summarizes the conditions evaluated to achieve NFs formation by electrospinning. Negative voltages were applied to the collector to direct the electrospinning jet towards the collector. Once stabilized under the efficient electrospinning conditions, NFs mats were collected over aluminum foil as membranes of ca. 20 × 15 cm^2^. The mats were dried under vacuum at 50 °C and their morphology analyzed by scanning electron microscopy (SEM).

**Figure 4:**
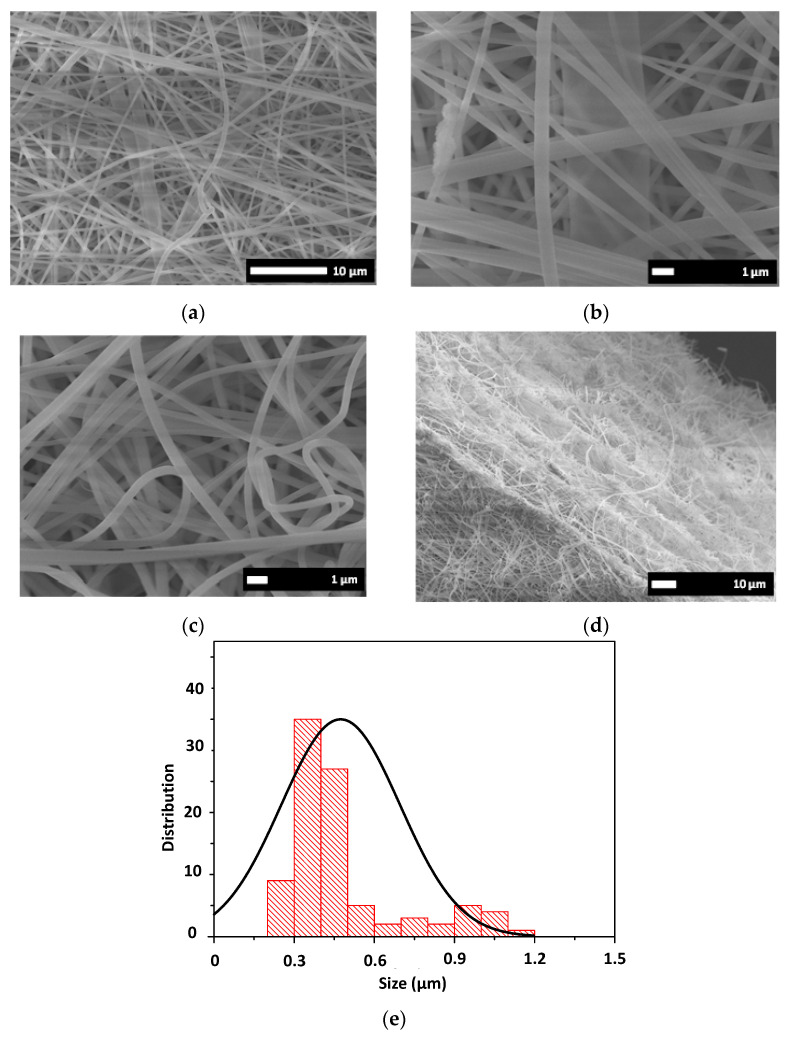
SEM images for the electrospun mat (NFm-**6**) formed from polymer blends PVP/TS-PIL **6** at different magnification. Surface view (**a**–**c**) and section view (**d**). Scale bar represent 10 µm (**a**,**d**) and 1 µm (**b**,**c**). (**e**) NFs size distribution.

Initially, electrospinning of a 65% *w/v* solution of polymer **6** in DMF was assayed. The material collected under these conditions was basically composed of beads mixed with some fibers (Figure S1). At the view of these initial results, a 50% weight/volume polymer blend solution formed by polyvinylpyrrolidone (PVP, Mw = 1,300,000 g/mol) and the polymer **6** in a 1:1 weight ratio in DMF was assayed. It has been reported that PVP-PILs blends can enable the production of NFs by electrospinning [18]. Using this solution and the conditions summarized in Table S1 the collection of NFs mats (NFm-**6**) was possible. Figure 4 shows some representative images of the morphologies observed for these electrospun mats. The PVP/**6** blend afforded a dense entanglement of bead-free fibers in most cases displaying a cylindrical form and with an average diameter of 474 nm (Figure 4a,c). The presence of some micro-ribbons instead of cylindrical fibers was also detected (Figure 4b). The width of these micro-ribbons was in the micrometer range (ca. 2.4 µm). The presence of these ribbons can be attributed to the association of some of the fibers [1]. The mat shows many interconnected NFs crossing in different direction defining a clear open porosity. The section of the NFs mat showed a thickness of ca. of 92 µm (Figure 4d).

The obtained NFm-**6** was also analyzed by ATR-FTIR showing the characteristics bands corresponding to both PVP and **6**. A complex broad band at 1750–1600 cm^−1^ was observed for the NFm-**6**, resulting from the overlapping of the individual bands corresponding to the carbonyl groups of the thiolactone and the amide fragments present in **6** and the amide group from PVP. The mat also showed the peak assignable to the amide II of **6**, which appeared red shifted (1553 cm^−1^ vs. 1550 cm^−1^ for **6** alone). The thiolactone C-S band at 920 cm^−1^ also confirmed the presence of **6** in NFm-**6**. The skeletal symmetric stretching of the imidazolium ring along with components assigned to CH_2_(N) stretching appeared in the mat at 1169 cm^−1^, while peaks at 1157 cm^−1^ and 1167 cm^−1^ were found for the corresponding original PILs. The peak for the -CH- wagging ν(C–N) of PVP for NFm-**6** mat also showed a shift to 1289 cm^−1^ from the value of 1287 cm^−1^ for pure PVP. All these red shifts suggest an interaction between PVP and **6** leading to an intimate blending through electrospinning. This interaction justifies the morphology of the NFs observed by SEM, where no signals of phase separation were appreciated [22].

### 3.3. Post-Functionalization of the NFs Mat

Once demonstrated the suitability of polymer **6** blended with PVP for the preparation of NFs mats, their solubility and the effect of different solvents was evaluated. The results obtained are summarized on Table S2. The mat was soluble in polar solvents (water and methanol) while being insoluble in apolar media such as toluene, hexane or diethyl ether. In these solvents, the material maintained their shape (1 × 1 cm mat pieces) once dried. However, when dichloromethane was tested the size of the mat was reduced from 1 × 1 cm to 0.3 × 0.2 cm also increasing its rigidity. Thus, apolar solvents such as toluene and diethyl ether were suitable solvent media for the modification and washing of these mats. Thus, the post-functionalization of **6** with different amines and acrylates and acrylamides was assayed as highlighted in Figure 1. In a first experiment, the modification of NFm-**6** was performed in the presence of an amine by suspending the mats in the corresponding liquid amine at 60 °C for 20 h. The film was then rinsed with toluene and the excess solvent evaporated at 50 °C until constant weight.

**Figure 5:**
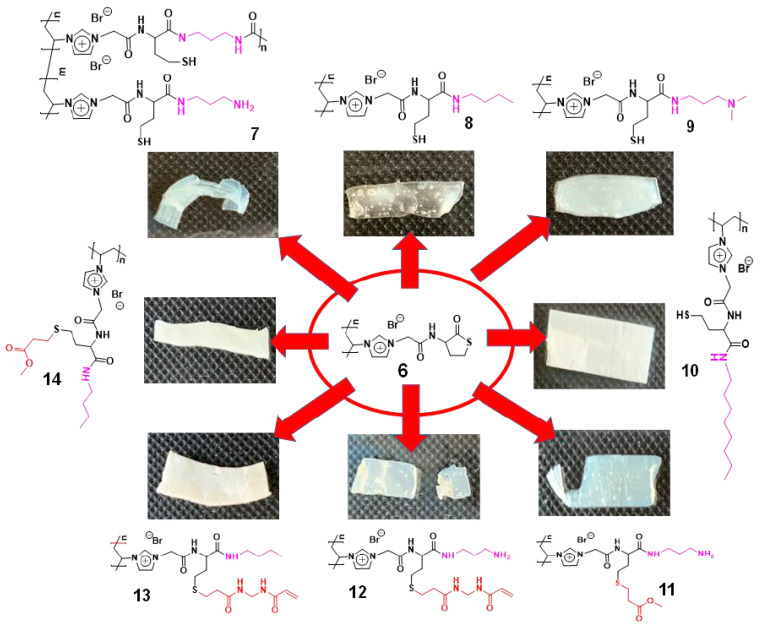
Post-modification of NFm-**6** using different reagents (amines and diamines). Pictures show the appearance of the mat after modification.

**Figure 6:**
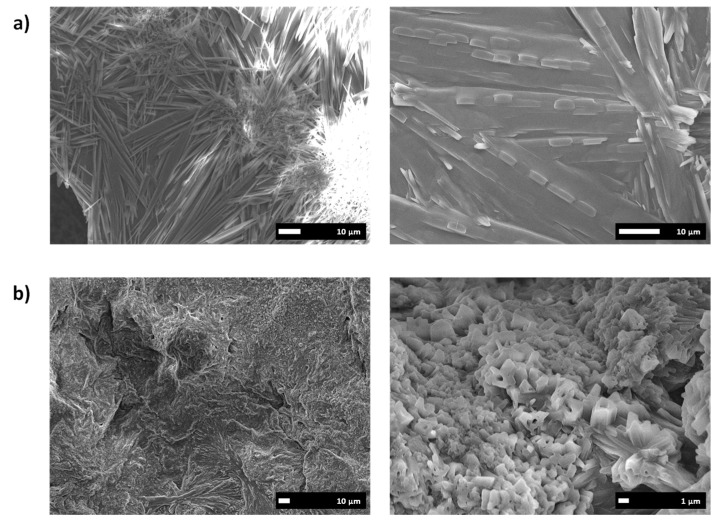
Sem pictures for the polymeric mats (**a**) Mat **7** (scale bars correspond to 10 µm) (**b**) Mat **8** (scale bars correspond to left: 10 µm, right: 1 µm).

Mat **7** was obtained by modification of NFm-**6** with 1,3-diaminopropane (Figure 5). The modification was confirmed by ATR-FTIR (Figure S3a) showing the disappearance of the peak at 920 cm^−1^ assignable to the C-S stretching of the thiolactone ring together with the presence of a strong band at 1557 cm^−1^ (amide II) assignable to the formation of new amide groups. The SEM pictures (Figure 6a) for mat **7** suggest that the post-modification of the membrane induced important changes on the morphology. The modified mat showed a complete porosity loss (Figure 6a vs. Figure 4). The di-topic nature of the amine can yield not only the aminolysis of the thiolactone but also the crosslinking of the polymeric NFs. An additional crosslinking of the NFs via S-S bridges is also possible. Thus, this double crosslinking mechanism by diamide and disulfide bridges led to strong NFs fusion inducing a complete loss of the initial structure. Similarly, the ATR-FTIR of the mat **8** confirmed the post-functionalization with butylamine (Figure 6b). SEM pictures also showed the fusion of NFs and the loss of the porosity. In this case, the change on morphology can be only due to the dynamic crosslinking and fusion of the NFs via S-S bridges (Figure 6b). It is noteworthy that the mats modified under these conditions changed their solubility properties, becoming insoluble in DMF.

**Figure 7:**
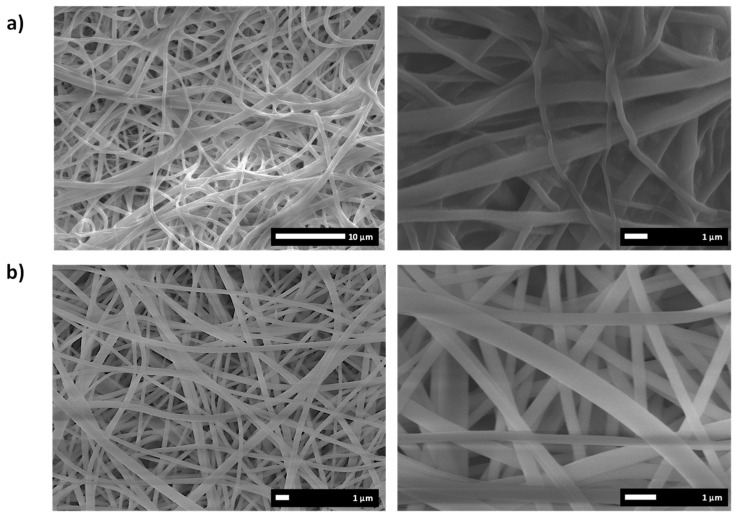
Sem pictures for the polymeric mats (**a**) Mat **9** (scale bars correspond to left: 10 µm, right: 1 µm) (**b**) Mat **10** (scale bars correspond to 1 µm).

ATR-FTIR spectra also demonstrated the successful modification of NFm-**6** with octylamine and 3-(dimethylamine)-1-propylamine affording mats **9** and **10** (Figures S5a and S6a). However, in this case, after the post-functionalization, the open porosity defined by the NFs in NFm-**6** was preserved (Figure 7a,b vs. Figure 4). The modification with 3-(dimethylamine)-1-propylamine produced an increase of the average diameter of the NFs from 474 nm (NFm-**6**) to 644 nm (Figure S5c). Similar results were also found for the mat modified with octylamine, although in this case with a slightly lower NFs size increase (545 nm, Figure S6c). Mats **9** and **10** were insoluble in DMF confirming the crosslinking of the NFs via S-S bridging of the thiols generated in-situ by the aminolysis of the thiolactone moieties. In comparison with the mat **8**, the larger hindrance introduce by the amine, can preclude, at some extent, the crosslinking via S-S bridge decreasing the deformation of the NFs and the loss of the open pore nanostructure of the mat.

**Figure 8:**
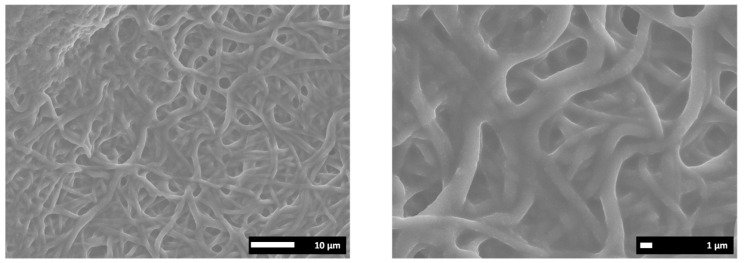
SEM pictures for the polymeric mat **11** (scale bars correspond to 10 µm (**left**), 1 µm (**right**)).

NFm-**6** could also be modified sequentially with 1,3-diaminopropane and methacrylate (mat **11**), which can react with the thiols obtained in-situ by aminolysis of the thiolactone group and avoiding additional crosslinking via S-S bridges and leading to two different levels of functionalization. The disappearance of the band at 920 cm^−1^ and the presence, among others, of a stronger amide II band at 1556 cm^−1^ confirmed the aminolysis, while the strong peak corresponding to the C=O of the ester at 1730 cm^−1^ suggested the success functionalization of the thiol (Figure S7a). SEM pictures of the modified mat showed that the NFs were fused and entangled together due to crosslinking induced by the diamine (Figure 8). Indeed, the NFs average size increased from 474 nm for the unmodified NFm-**6** to 922 nm. However, in this case, the addition of the acrylate avoided the crosslinking via S-S bridges allowing tus o keep the nanostructure of open pores of the mat (Figure 8 vs. Figure 4), although the porosity was greatly reduced in comparison with the unmodified NFm-**6**.

In the search of the additional methodologies allowing the simultaneous modification and crosslinking of the NFs while maintaining the nanostructure and open porosity the second level modification with *N,N*′-methylenebis(acrylamide) was evaluated. The bis-acrylamide, in principle, can react through a Michael reaction with the generated thiol groups providing an additional possibility for crosslinking. Two different amines, 1,3-diaminopropane and butyl amine, were assayed for the first level of functionalization. The first combination can lead to crosslinking both through the aminolysis and through thiol modification, while in the case of butyl amine the crosslinking would be only due to the thiolene click reactions. The modification was followed by ATR-FTIR and afforded the modified mats **12** and **13** (Figures S8a and S9a). The mat modified with 1,3-diaminopropane and bisacrylamide showed an almost completely loss of the porosity (Figure S8b). However, thick fused cylindrical NFs were still observable. This change in the morphology can be attributed to the extensive crosslinking provided by the bifunctional modifiers. When the modification was performed with butyl amine, instead of 1,3-diaminopropane, a complete loss of the NFs morphology was appreciated (Figure S9b). This reveals that S-S bridging represents a more efficient mechanism for NFs fusion with the corresponding loss of the original nanostructure.

Finally, the modification of NFm-**6** was performed with butyl amine and methyl methacrylate to minimize the crosslinking of the fibers. The modification was successfully achieved as the ATR-FTIR showed again the band for the ester group at 1738 cm^−1^ and a strong amide II band at 1557 cm^−1^ along with the total disappearance of the C-S peak of the thiolactone group (Figure S10a). The mat after the modification showed entangled cylindrical NFs with an average size of 710 nm, thus preserving the essential nanostructure but achieving a reduction in the porosity in comparison with the morphology observed for unmodified NFm-**6**.

In summary, the presence of the thiolactone unit allows the chemical post-modification of the NFs, although the resulting nanostructure of mats obtained was highly dependent on the nature of the modifiers.

### 3.4. Application of the NFs Mat to Sensing

In the search of possible applications for the materials here prepared, a series of proof-of-concept studies were carried out. Materials composed by NFs mats have found promising applications as sensing devices [23], for instance, for a fast and low-cost detection of volatile amines [24]. Natural volatile amines, which are often toxic, can be present in our daily life (i.e., chemical manufacturing, agriculture and farming, release by rotten food or exhalation under certain medical conditions or diseases, etc.) [25,26]. In the same way, ILs and related materials have shown to be of interest in optical sensing technologies [27,28]. Different sensing principles have been reported for volatile amines detection. Among them, the use of pH colorimetric sensors, which change their color upon contact to the volatile amine, represents a facile, cost-effective, and non-power operated material for gas amine detection [29].

**Figure 9:**
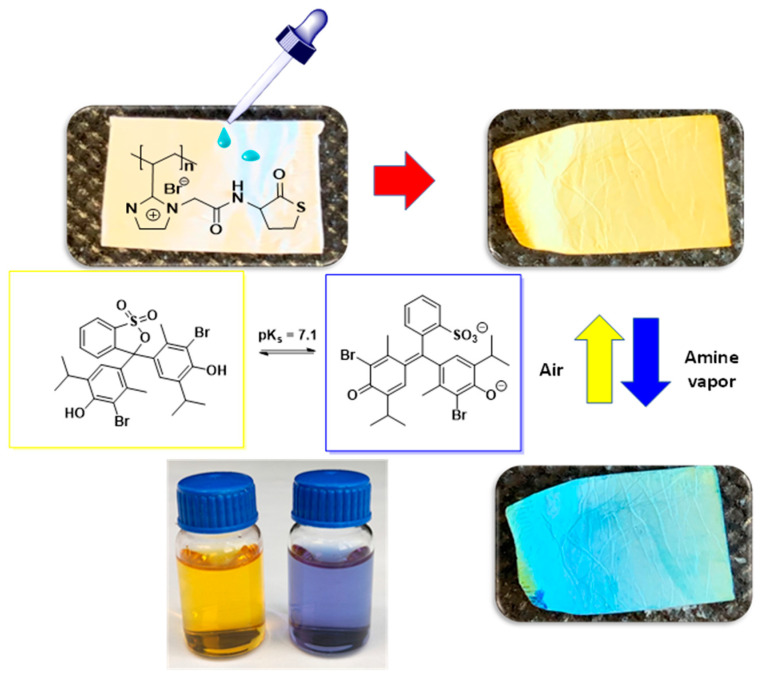
Naked-eye detection of a volatile amine (NH_3_) using NFm-**6** doped with bromothymol blue (BTB).

For this purpose, NFm-**6** films were impregnated with a solution of bromothymol blue (BTB) in toluene (2 mg in 10 mL). The films were dried under vacuum until constant weight showing a yellow color due to the presence of the BTB in its acid form (Figure 9). To evaluate the ability of these mats as volatile amine sensor, they were exposed to the vapors of different amines (NH_3_, methylamine, butylamine, iso-propylamine and piperidine). In all the cases, the NFs mats experienced, independently of the amine nature, a color change from yellow to blue in less than a few seconds, easily observable by the naked eye (see Video S1). The change agrees with those for aqueous solutions of BDB thar are yellow at pH < 7, green under neutral pH and blue at pH > 7. The color change was reversible, and the mats could be used in consecutives cycles of air-amine exposure (see Video S1). It is also noteworthy that the films also responded to the presence of acid gases (HCl) experimenting film shrinking and a color change to violet. The change in size can be explained through an increase in the ionic interactions in the fibers.

The colorimetric behavior of NFm-**6** was compared, under the same conditions, with that of films prepared by casting from and identical polymeric blend. The sensor obtained by electrospinning, when exposed to the volatile amine, showed a faster and more intense color change than the mat prepared by casting (Figure S11 and Videos S1–S3). Thus, NFm-**6** is able to break the so-called “trade-off” rule in sensing harnessing high sensitivity and fast response simultaneously in comparison with the membrane obtained by casting. The good response relies on the open and well-defined porous structure defined by the NFs (Figure 3) allowing a fast diffusion of the target analyte into the membrane and enhancing its interaction with the sensing unit.

These initial results demonstrated that NFs mats obtained from TS-PILs **6** are a suitable platform for sensing application as: (i) the TS-PIL units do not react or interfere with the sensing dye, (ii) the polymer provides a uniform background not interfering in the color change, (iii) the mats present good surface properties, allowing the colorimetric sensor to spread uniformly, which is reflected in the uniform mat color, and (iv) the NFs structure obtained by electrospinning render a microstructure with high surface area enhancing the diffusion of the analyte to the sensing unit and leading to a fast response.

### 3.5. Application of the Fiber-Mats for Catalysis

Electrospun NFs presenting catalytic units have demonstrated great potential in a variety of catalytic application [30]. The large surface area and optimal surface chemistry of electrospun nanofibers greatly enhance the catalyst–support interaction and can improve their activity, selectivity, stability, and reusability. Although NFs mats obtained by electrospinning offers very attractive properties for the development of catalytic systems, there is not a single application, as far as we know, of electrospun NFs mats based on PILs and used in catalysis.

**Scheme 2:**
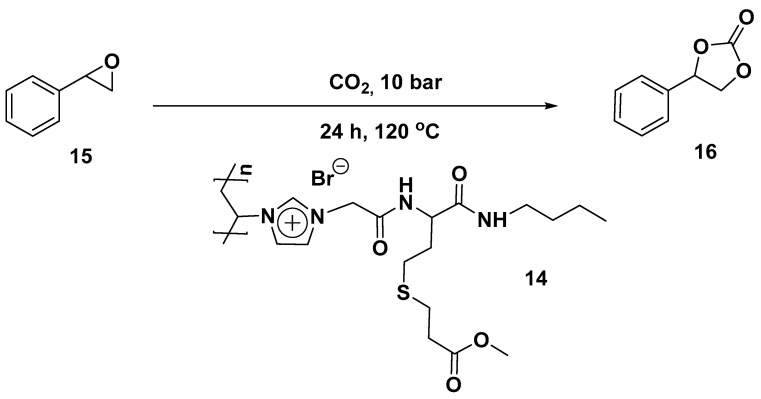
Cycloaddition reaction of CO_2_ to styrene epoxide catalyzed by **14.**

Advanced materials based on PILs have been used as efficient systems for the capture, activation and conversion of CO_2_ [31]. For instance, different polymers containing IL-like moieties have been reported as efficient catalysts for the cycloaddition reaction of CO_2_ with various epoxides to generate cyclic carbonates, which is one of the most promising and efficient approaches for CO_2_ fixation [32]. In this context, the modified mat **14** was studied for the catalytic reaction of styrene oxide with CO_2_. In general, this process requires harsh experimental conditions (temperature > 130 °C and pressure > 10 bars) to lead to the corresponding carbonates. When the mat **14** was tested as a heterogeneous catalyst for this reaction (Scheme 2) 44% of conversion of the epoxide was observed with full selectivity to the corresponding carbonate at 120 °C and 10 bar after 24 h. This is a remarkable result if we consider the bromide content, as this is expected to be the nucleophilic anion catalyzing the reaction, which was only 0.03% mol Br/substrate. This represents a notable TON of 1244.

## 4. Conclusions

We have demonstrated that NFs mats derived from the TS-PILs based on homocysteine thiolactone can be obtained by electrospinning them as blends with PVP. The presence of this functional moiety allowed the post-functionalization of these mats through the aminolysis of the thiolactone ring in the presence of an amine that can be followed by a thiol–alkene “click” reaction. Under controlled experimental conditions the modification can be performed introducing different functionalization and crosslinking of the NFs. The morphology of the modified mats was highly dependent of nature of the modifiers. Different conditions were stabilized for the modification of the mat while maintaining the nanostructure obtained by the electrospinning. Initial studies suggest that the NFs based on these functionalized PILs can be used in both sensing and catalytic applications.

## Data Availability

Not applicable.

## Supplementary Materials

The following are available online at https://drive.google.com/file/d/12oPPZsJbuGy0o07QhWyr92Iv_spenN_l/view?usp=drive_web. Figure S1: Images of fibers with the formation of beads obtained by electrospinning of polymer 6 (65 % w/v). (a) Optical microscope imagen, 10 x / 0.25 mm. (b) SEM imagen, scale bar represents 10 μm, Figure S3: (a) Comparison of the ATR-FTIR spectra for NFm-6 and 7. (b) Optical images for mat 7 after modification. and structure, Figure S5: (a) Comparison of the ATR-FTIR for the NFs mat 6 and 9. (b) Optical imagine of the mat 9 after modification and structure. (c) NFs size distribution., Figure S6: (a) Comparison of the ATR-FTIR for the NFs mat 6 and 10. (b) Optical imagine of the mat 10 after modification and structure. (c) NFs size distribution, Figure S7: (a) Comparison of the ATR-FTIR for the NFs mat 6 and 11. (b) Optical imagine of the mat 11 after modification and structure. (c) NFs size distribution, Figure S8: (a) Comparison of the ATR-FTIR for the NFs mat 6 and 12. (b) Optical imagine of the mat 12 after modification and structure. (c) SEM of the polymeric mat 12, scale bars correspond to 10μm, Figure S9: (a) Comparison of the ATR-FTIR for the NFs mat 6 and 13. (b) Optical imagine of the mat 13 after modification and structure. (c) SEM of the polymeric mat 13, scale bars correspond to10μm, Figure S10: (a) Comparison of the ATR-FTIR for the NFs mat 6 and 14. (b) Optical imagine of the mat 14 after modification and structure. (c) SEM of the polymeric mat 14, scale bars correspond to 10μm (d) NFs size distribution, Figure S11: Comparison of color changing of the polymer blend PVP/TS-PIL 6 obtained by (a) casting or by (b) electrospinning in presence of the NH_3_ vapor, Figure S14: Spectrum of polymer 6. (a) ^1^H NMR spectra in D_2_O and (b) ^13^C NMR Spectra in D_2_O, Table S1: Electrospinning parameters used to fabricate NFs mats., Table S1: Solubility evaluation of the NFs mat obtained from PVP/TS-PIL 6, Video S1: Colorimetric changes of NFm-6 with butylamine, Video S2: Colorimetric changes of NFm-6 with different amines, Video S3: Colorimetric changes of NFm-6 with ammonia.
